# Comprehensive evaluation of coagulability using thromboelastography in four patients with essential thrombocythemia

**DOI:** 10.1186/s40981-025-00789-6

**Published:** 2025-05-17

**Authors:** Shuji Kawamoto, Tsuguhiro Matsumoto, Yohei Chiwata, Chikashi Takeda, Eriko Kusudo, Moritoki Egi

**Affiliations:** Department of Anesthesia, Kyoto University Hospital, 54 Shogoin- Kawahara-Cho, Sakyo-Ku, Kyoto, 606-8507 Japan

**Keywords:** Essential thrombocythemia, Coagulation, Platelet, Thromboelastography

## Abstract

**Background:**

Essential thrombocythemia (ET) is a myeloproliferative neoplasm characterized by increased platelet count and risk of thrombosis and bleeding, which necessitates careful perioperative management. However, there are no standardized guidelines for perioperative antithrombotic therapy, and optimal preoperative evaluation remains unclear. In this report, we evaluate the utility of thromboelastography (TEG®6 s) for assessing coagulation and platelet function in ET patients undergoing surgery.

**Case presentation:**

Four ET patients (platelet counts: 289,000–833,000/µL) underwent thromboelastography at anesthesia induction. Two had normal coagulation, while two had a hypercoagulable state undetected by conventional tests. Hypercoagulability was observed in patients who discontinued anticoagulants or antiplatelets preoperatively.

**Conclusions:**

Thromboelastography identified thrombotic tendencies not evident with conventional coagulation tests, suggesting its potential for perioperative risk assessment in ET patients. This approach may improve individualized coagulation management beyond use of platelet counts and standard tests. Further studies are needed to establish the role of thromboelastography in optimizing perioperative antithrombotic strategies.

## Background

Essential thrombocythemia (ET) is a type of myeloproliferative neoplasm characterized by clonal proliferation of hematopoietic stem cells, which leads to an abnormal increase in platelet count. According to the 2016 revision of the World Health Organization (WHO) classification, the diagnosis of ET is based on sustained thrombocytosis (platelet count ≥ 450 × 10⁹/L), characteristic megakaryocytic proliferation in the bone marrow, exclusion of other myeloid neoplasms, and the presence of clonal markers such as mutations in Janus kinase 2 (JAK2), calreticulin (CALR), or the myeloproliferative leukemia virus oncogene (MPL) [1]. In ET, both thrombosis and bleeding tendency are clinical concerns. Patients older than 60 years of age or with a history of thrombosis are considered at high risk for thrombosis, and platelet activation is enhanced and the risk of thrombus formation is increased, especially in patients with ET with JAK 2 mutations [2, 3]. Conversely, bleeding tendency in ET is associated with decreased von Willebrand factor (VWF) activity [4]. When the platelet count exceeds 1,000,000/μL, large-molecular-weight multimers of VWF are consumed. This impairs primary hemostasis and increases the risk of bleeding [5], with epidemiological studies showing bleeding events in the range of 4–10% in patients with ET [6, 7]. Thus, in perioperative management of these patients, it is crucial to assess thrombotic and bleeding risks and select appropriate antithrombotic therapy. In a systematic review of abdominal surgery in patients with ET, Zhu et al. found a high risk of perioperative thrombosis and bleeding, necessitating meticulous coagulation management [8]. However, evidence for such perioperative management in patients with ET remains limited, with many unresolved challenges in thrombotic and bleeding management postoperatively.

Thromboelastography has recently been reported to be useful for evaluating thrombotic tendencies and hemostatic abnormalities that cannot be fully captured in conventional coagulation tests. Here, we present findings from four patients with ET who were scheduled for surgery. To evaluate the effectiveness of preoperative antithrombotic therapy, we utilized the TEG®6 s thromboelastography system at induction of general anesthesia, with assessment of coagulability and platelet function using Global Hemostasis and PlateletMapping®.

## Case presentation

Written consent was obtained from all the patients for publication of this case series. The patient characteristics are shown in Table 1 and the details of preoperative treatment and assessment are summarized in Table 2. Preoperative treatment was determined by the attending hematologist or primary physician based on each patient’s clinical background. At our hospital, anticoagulants and antiplatelet agents are generally managed according to standard clinical practices commonly followed in perioperative care. Final decisions are made individually considering surgical and patient-specific risks. Some treatments therefore differed from these guidelines, reflecting real-world clinical practice. In all cases, blood samples for thromboelastography were collected from the patients’ peripheral vein prior to the induction of anesthesia.

**Table 1:** Background of the four cases

Case	Age (years)	Sex	ET duration (years)	Preoperative platelet count (× 10^3^/μL)	Preoperative PT-INR	Preoperative APTT (seconds)	Preoperative fibrinogen (mg/dL)	JAK2 mutation	History of thrombosis
1	60 s	Female	5	726	1.05	30.2	310	-	-
2	80 s	Female	2	577	1.30	31.8	482	+	+
3	70 s	Male	5	833	1.22	34.8	513	+	-
4	80 s	Female	3	289	0.97	37.2	301	+	-

*ET* Essential thrombocythemia, *PT-INR* Prothrombin time-international normalized ratio, *APTT* Activated partial thromboplastin time, *JAK2* Janus kinase 2

**Table 2:** Preoperative treatment and evaluation in the four cases

Case	Preoperative treatment	Discontinuation or continuation of preoperative antithrombotic treatment	Risk classification for thrombosis (Barbui et al. [3])	TEG®6 s characteristics
1	Hydroxycarbamide (1000–1500 mg/day), Aspirin (81 mg/day)	Aspirin continued	Intermediate	Normal range
2	Ruxolitinib, Edoxaban (30 mg/day)	Edoxaban stopped 1 day before surgery	High	Hypercoagulable state: High CK, CRT, CKH, CFF, HKH, ActF
3	Anagrelide, Hydroxycarbamide (1000 mg/day), Aspirin (81 mg/day)	Aspirin stopped 3 days before surgery	High	Hypercoagulable state: High CK, CRT, CKH, CFF, HKH, ActF
4	Hydroxycarbamide (500 mg/day), Limaprost alfadex	Limaprost alfadex continued	High	Normal range

Thrombotic risk classification was based on the revised IPSET-thrombosis model [3], which defines four risk levels: very low (age ≤ 60, JAK2-wild type, no thrombosis), low (age ≤ 60, JAK2-mutation), intermediate (age > 60, JAK2-wild type), and high (thrombosis history or age > 60 with JAK2 mutation)

*CK* Citrate kaolin, *CRT* Citrated rapid TEG, *CKH* Citrate kaolin with heparinase, *CFF* Citrated functional fibrinogen, *HKH* Heparinized kaolin with heparinase, *ActF* Activator F

### Case 1

**Fig. 1:**
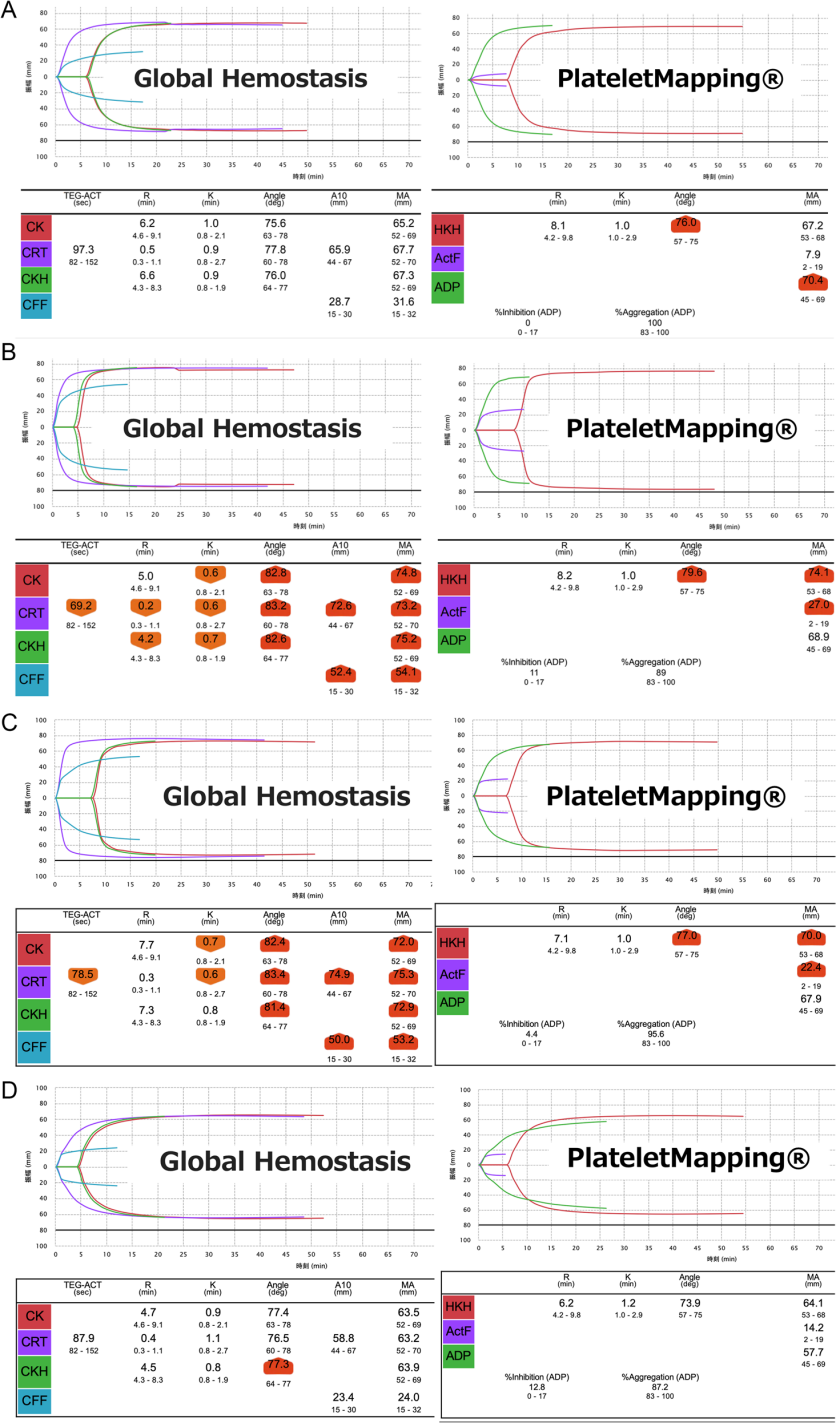
TEG®6 s Global Hemostasis and PlateletMapping® results. A. Case 1, B. Case 2, C. Case 3, D. Case 4. Highlighted areas indicate abnormal values. The normal range of each parameter is shown at the bottom of the values. CK: citrate kaolin, CRT: citrated rapid TEG, CKH: citrate kaolin with heparinase, CFF: citrated functional fibrinogen, ActF: activator F, HKH: heparinized kaolin with heparinase, ADP: adenosine diphosphate. Shortened reaction time (R) and clot formation time (K), along with increased alpha angle (angle) and maximum amplitude (MA), are indicative of a hypercoagulable state. In this figure, Cases 2 and 3 show such hypercoagulable profiles, whereas Cases 1 and 4 exhibit values within the normal range, suggesting balanced coagulation

A woman in her 60 s was diagnosed with JAK2-negative ET five years ago. She was scheduled for video-assisted thoracoscopic right lung partial resection for metastatic lung tumors from breast cancer. However, surgery was postponed due to a transient peak platelet count of 2,000,000/µL. After alternate-day administration of a cytoreductive agent, hydroxycarbamide (1,000–1,500 mg/day), her platelet count decreased to 726,000/µL over six months, allowing the procedure to proceed. Aspirin (81 mg/day) was continued without interruption before surgery. Preoperative tests showed a prothrombin time-international normalized ratio (PT-INR) of 1.05, activated partial thromboplastin time (APTT) 30.2 s, fibrinogen 310 mg/dL, D-dimer 0.3 mg/dL, and VWF activity of 133% (reference range: 50–150%). TEG®6 s Global Hemostasis and PlateletMapping® assessments performed after induction of general anesthesia were within normal ranges (Fig. 1A). The surgery lasted 1 h and 6 min, the total anesthesia time was 3 h and 8 min, and estimated blood loss was 160 mL. There were no perioperative complications, and the patient recovered uneventfully and was discharged home on postoperative day 4.

### Case 2

A woman in her 80 s was diagnosed with JAK2-positive ET two years ago and had been taking the JAK inhibitor, ruxolitinib. Although ruxolitinib is mainly used for myelofibrosis and polycythemia vera, it was prescribed by the attending hematologist. The diagnosis of ET was confirmed based on WHO criteria. She had a history of incidental pulmonary embolism and a descending aortic thrombus and was receiving a direct factor Xa inhibitor, edoxaban (30 mg/day). Edoxaban was discontinued one day before surgery for transurethral bladder tumor resection. Preoperative tests showed a platelet count of 577,000/µL, PT-INR 1.30, APTT 31.8 s, fibrinogen 482 mg/dL, and D-dimer 0.5 mg/dL. TEG®6 s showed significant hypercoagulability, with increased maximum amplitude (MA) of citrated rapid TEG (CRT) and heparinized kaolin with heparinase (HKH), which indicate comprehensive hemostatic performance, and of citrated functional fibrinogen (CFF) and activator F (ActF), which correlate with fibrinogen levels (Fig. 1B). The surgery lasted 1 h and 11 min, with an anesthesia time of 2 h and 7 min, and minimal blood loss. Edoxaban was restarted the day after surgery. There were no perioperative complications, and the patient recovered uneventfully and was discharged home on postoperative day 2.

### Case 3

A man in his 70 s was diagnosed with JAK2-positive ET five years ago. Due to persistently elevated platelet counts (> 1,000,000/µL), he had been receiving a combination of a platelet production inhibitor, anagrelide, and hydroxycarbamide (1,500 mg/day). He underwent laparoscopic left nephrectomy, with preoperative tests showing a platelet count of 833,000/µL, PT-INR 1.22, APTT 34.8 s, fibrinogen 513 mg/dL, and D-dimer 0.8 mg/dL. Aspirin (81 mg/day) was discontinued 3 days before surgery. This was based on institutional practice for moderate-risk procedures to reduce bleeding risk. TEG®6 s revealed significantly elevated MA of CRT, HKH, CFF and ActF, confirming a pronounced hypercoagulable state (Fig. 1 C). Surgery lasted 4 h and 55 min, with an anesthesia time of 7 h and 16 min, and minimal blood loss. The patient had no perioperative complications and was discharged after an uneventful recovery.

### Case 4

A woman in her 80 s was diagnosed with JAK2-positive ET three years ago. After starting hydroxycarbamide (500 mg/day), her platelet count stabilized at approximately 300,000/µL. She discontinued aspirin due to pruritus and was not on antiplatelet therapy. She was taking a prostaglandin E_1_ derivative, limaprost alfadex, for lumbar spinal canal stenosis and continued this medication during scheduled laparoscopic right inguinal hernia repair. Preoperative tests showed a platelet count of 289,000/µL, PT-INR 0.97, APTT 37.2 s, and fibrinogen 301 mg/dL. TEG®6 s results were within the normal range (Fig. 1D). The surgery lasted 1 h and 6 min, with an anesthesia time of 2 h and 5 min, and minimal blood loss. There were no perioperative complications and the patient was discharged on postoperative day 2.

## Discussion

Coagulation and platelet function were evaluated using preoperative thromboelastography in four patients with ET who were scheduled for surgery. The patients showed significant variation in thromboelastography findings, which may have been influenced by ET-specific variations in coagulation capacity and by perioperative antithrombotic therapies. Patients who discontinued anticoagulants or antiplatelets preoperatively (Cases 2 and 3) exhibited marked hypercoagulable tendencies that were not detected by conventional coagulation tests or platelet counts alone, while those who continued antithrombotic agents (Cases 1 and 4) had relatively normal thromboelastography results (Fig. 1 A-1D). The effects of hydroxyurea, anagrelide, and ruxolitinib on coagulation may vary and could influence thromboelastography results.

Thrombotic risk in patients with ET is not solely determined by platelet count, but also by platelet dysfunction, coagulation factor imbalances, and fibrinolysis changes [9, 10]. The 2024 Guidelines for Hematologic Malignancies of the Japanese Society of Hematology recommend thrombotic risk assessment and tailored antithrombotic therapy for patients with ET [11]. Patients aged 60 or above or those with a history of thrombosis are considered at high risk for thrombosis and are indicated for antithrombotic therapy [11]. In the risk classification for thrombosis described by Barbui et al. [3], which is also in the guidelines and further incorporates the presence or absence of JAK2 mutations, Cases 2, 3 and 4 are classified as high risk (Table 2). Although the target platelet count is generally set at 400,000 to 600,000/μL in routine practice, its role as a risk factor remains inconclusive [11].

Thromboelastography provides a comprehensive assessment of platelet function, fibrin formation, and fibrinolysis, compared to platelet count and standard coagulation tests (PT-INR, APTT, fibrinogen) [12–14]. Case 2 showed hypercoagulability despite a platelet count below 600,000/µL, with abnormally high CFF and ActF, reflecting the effect of fibrinogen, which plays a key role in hemostasis. In patients with ET, increased fibrinogen receptor (GPIIb/IIIa) expression occurs with increased platelet counts, which may contribute to hypercoagulability [15, 16]. Thromboelastography may show hypercoagulation in cases with lower platelet counts, indicating that these counts and the fibrinogen level alone do not fully capture the coagulation state. Furthermore, edoxaban was withdrawn preoperatively in Case 2, but thromboelastography still showed a marked tendency toward hypercoagulability. In this case, a hypercoagulable state was observed after standard preoperative withdrawal of edoxaban, suggesting that thromboelastography may help identify patients who require more individualized perioperative management.

In Case 3, aspirin was discontinued and a pronounced hypercoagulable state was observed. The effect of aspirin on platelet function via the arachidonic acid pathway cannot be evaluated using ADP-aggregation and ADP-MA, which assess ADP-receptor responsiveness. Therefore, the assessment relies on parameters such as CRT and HKH, which reflect overall coagulation capacity. In such patients, it may be appropriate to continue with or introduce antithrombotic therapy preoperatively.

VWF abnormalities also influence ET pathophysiology. Increased platelet counts can lead to VWF adsorption onto platelet surfaces, reducing the VWF plasma concentration and resulting in acquired von Willebrand disease [17]. VWF deficiency contributes to bleeding tendency, but thrombotic risk is influenced by additional factors, making single-marker assessment inadequate [17]. Although bleeding risk is generally higher in ET patients with platelet counts exceeding 1,000,000/µL, it may also occur at lower counts due to platelet dysfunction or VWF abnormalities.

The results in our cases suggest that a comprehensive evaluation of coagulation using thromboelastography may contribute to individualized risk assessment and appropriate adjustment of antithrombotic therapy in perioperative ET management, rather than relying solely on platelet counts, fibrinogen, and conventional coagulation tests. Despite appropriate antithrombotic therapy based on risk stratification, thrombotic events may still occur in ET patients. Tefferi et al. reported a cumulative incidence of approximately 12% over 6 years in high-risk patients [18]. Therefore, thromboelastography may serve as a supplementary tool to detect residual hypercoagulability and support more individualized perioperative management. The 2024 Japanese guideline is consistent with international recommendations for thrombotic risk classification but does not provide detailed instructions for perioperative antithrombotic management [11, 19]. Therefore, treatment is often determined by the attending physician based on individual patient and surgical factors. In this context, thromboelastography may be useful for assessing thrombotic risk following anticoagulant discontinuation, as a valuable tool for guiding personalized perioperative anticoagulant management. However, a limitation of this report is that thromboelastography was performed only at a single time point—prior to induction of general anesthesia—and not at multiple perioperative stages. Consequently, we were unable to evaluate dynamic changes in coagulation status before and after the discontinuation or continuation of antithrombotic therapy. Despite this limitation, the thromboelastography findings provided clinically meaningful insights into each patient’s coagulation state immediately before surgery. Notably, in Cases 2 and 3, a hypercoagulable state was detected even after antithrombotic agents had been discontinued, which would have been difficult to identify using conventional coagulation tests alone. These results suggest that even single-point thromboelastography may support individualized perioperative management; however, this case series does not provide data to determine whether thromboelastography can guide the decision to discontinue or continue antithrombotic therapy, as dynamic changes in response to treatment were not evaluated. Future studies should consider serial thromboelastography assessments at multiple perioperative time points to better understand the impact of antithrombotic therapy modifications.

## Data Availability

All data generated or analyzed in the study are included in this article.

## Abbreviations

ET: Essential thrombocythemia
WHO: World Health Organization
JAK: Janus kinase
CALR: Calreticulin
MPL: Myeloproliferative leukemia virus oncogene
VWF: Von Willebrand factor
PT-INR: Prothrombin time-international normalized ratio
APTT: Activated partial thromboplastin time
CK: Citrate kaolin
MA: Maximum amplitude
CRT: Citrated rapid TEG
CFF: Citrated functional fibrinogen
ActF: Activator F
HKH: Heparinized kaolin with heparinase
ADP: Adenosine diphosphate
